# Caltech Conte Center, a multimodal data resource for exploring social cognition and decision-making

**DOI:** 10.1038/s41597-022-01171-2

**Published:** 2022-03-31

**Authors:** Dorit Kliemann, Ralph Adolphs, Tim Armstrong, Paola Galdi, David A. Kahn, Tessa Rusch, A. Zeynep Enkavi, Deuhua Liang, Steven Lograsso, Wenying Zhu, Rona Yu, Remya Nair, Lynn K. Paul, J. Michael Tyszka

**Affiliations:** 1 Division of Humanities and Social Sciences, California Institute of Technology, 1200 E California Boulevard, Pasadena, CA 91125 USA; 2 Department of Psychological and Brain Sciences, University of Iowa, 340 Iowa Ave, Iowa City, IA 52242 USA; 3 Division of Biology and Bioengineering, California Institute of Technology, 1200 E California Boulevard, Pasadena, CA 91125 USA; 4 Schmid College of Science and Technology, Chapman University, University Drive, Orange, CA 92866 USA; 5 MRC Centre for Reproductive Health, The University of Edinburgh, 47 Little France Crescent, Edinburgh, EH16 4TJ UK

**Keywords:** Cognitive neuroscience, Neuroscience

## Abstract

This data release of 117 healthy community-dwelling adults provides multimodal high-quality neuroimaging and behavioral data for the investigation of brain-behavior relationships. We provide structural MRI, resting-state functional MRI, movie functional MRI, together with questionnaire-based and task-based psychological variables; many of the participants have multiple datasets from retesting over the course of several years. Our dataset is distinguished by utilizing open-source data formats and processing tools (*BIDS, FreeSurfer, fMRIPrep, MRIQC*), providing data that is thoroughly quality checked, preprocessed to various extents and available in multiple anatomical spaces. A customizable denoising pipeline is provided as open-source code that includes tools for the generation of functional connectivity matrices and initialization of individual difference analyses. Behavioral data include a comprehensive set of psychological assessments on gold-standard instruments encompassing cognitive function, mood and personality, together with exploratory factor analyses. The dataset provides an in-depth, multimodal resource for investigating associations between individual differences, brain structure and function, with a focus on the domains of social cognition and decision-making.

## Abstract (summary)

**Table:** 

Measurement(s)	Blood Oxygen Level-Dependent Functional MRI • brain • psychological measurement
Technology Type(s)	functional magnetic resonance imaging • 3 T MRI scanner • behavioral assessments
Sample Characteristic - Organism	Homo sapiens
Sample Characteristic - Location	United States of America

## Background & Summary

Investigating brain-behavior relationships and their individual differences requires multimodal data that include at least neural data (typically structural or functional magnetic resonance imaging data, structural MRI (sMRI) or functional MRI (fMRI)) together with behavioral data (typically questionnaire-based scores). Traditionally, such data have been acquired in individual studies, often with modest sample sizes, and focused on a specific research question. Several more recent datasets combine neuroimaging and behavioral data in larger samples with broader but shallow coverage of cognitive domains; a few datasets also provide exceptionally dense data with deep phenotyping, but in very small samples^1,2^. For instance, the UK Biobank^3^ provides very broad behavioral data together with MRI and genetic data on 500,000 subjects – a resource that has been utilized by over 20,000 researchers to date and has yielded a number of important findings^4,5^. At the opposite extreme, dense longitudinal rest-state fMRI acquired on a single individual showed that functional brain networks are more fine-grained than originally thought^1^. For many large databases, sample sizes are now becoming sufficiently large that nonlinear modeling (e.g., with deep learning) is becoming possible to apply to brain-behavior relationships^6^. However, breadth across variables and large samples typically come at the expense of shallow assessment of most behavioral variables, and often limited quality control over individual neuroimaging data. For instance, cognitive variables like intelligence are assessed with short tasks or questionnaires, rather than gold-standards in the field such as the Wechsler Adult Intelligence Scale^7^, which may result in poor precision as well as low construct validity. More comprehensive psychological assessment is provided in the Human Connectome Project, (HCP), which also provides high-resolution structural and functional neuroimaging data^8^ and remains a multimodal resource generating a large number of novel discoveries (see https://www.humanconnectome.org/study/hcp-young-adult/publications). However, across datasets, there is a tradeoff between in-depth and/or quality-checked data, on the one hand, versus sample size and domain breadth, on the other hand. For instance, social cognition is multifaceted and complex. Thus it can only be adequately assessed with a variety of measures to describe individual functioning (and potential individual differences therein). In addition, a major practical limitation for users is that databases generally provide only specific neuroimaging formats and processing steps, which often become outdated or require further conversion and processing before analyses can be applied.

Our dataset lies intermediate in the space of databases outlined above: a medium-sized sample (n = 117) but with exceptional quality control, range of data types, accessibility and ease of usability. Manually quality-checked structural and denoised functional (resting-state and movie) MRI data are organized in BIDS^9^ and are provided with quality metrics and in multiple processed formats (including individual native space and multiple standard template spaces). We use standardized preprocessing and quality control tools, such as fMRIPrep^10^, FreeSurfer^11^, and MRIQC^12^. Behavioral data encompass comprehensive metrics on intelligence, personality, mood, and social cognition. A subset of the subjects were retested over months-years. All data were collected by the NIMH-funded Caltech Conte Center (http://conte.caltech.edu) and are tailored towards investigations of social cognition and decision-making. The precision with which cognitive processes can be estimated usually demands longer tasks or questionnaires, and/or the extraction of latent factors across multiple observed measures, both of which we provide here. As detailed in Table 1 below, the dataset includes a variety of gold-standard psychological state and trait variables relevant to social decision-making. For example, intelligence provides an important continuous variable that could be used as a covariate in all analyses. Likewise measures of depression, stress, valenced mood and anxiety may be used as covariates or utilized for formulating exclusionary criteria. The dataset also includes trait-level questionnaire-based measures that specifically address social decision-making and behavior. For example, several of our measures are commonly used to assess traits related to autism spectrum disorder (e.g. Empathizing Quotient^13^, Systematizing Quotient^14^, and Social Responsiveness Scale^15^), the social network index has been widely used as a proxy for social connectedness in real life, and the 16PF personality questionnaire provides a fine-grained assessment of personality traits related to social engagement from which the standard “Big-Five” personality factors can be easily derived. Especially notable is the Mayer-Salovey-Caruso Emotional Intelligence Test^16^, a comprehensive and time-intensive collection of questionnaire-based and task-based measures that index multiple facets of social and emotional ability. Taken together, this array of behavioral measures provides a particularly rich assessment of individual differences relevant to social decision-making, and item-level data availability permits researchers to explore additional structure. We have included an exploratory factor analysis to showcase how the included measures and their loadings on potentially underlying factors can be used to leverage the richness of this new dataset towards novel questions in human cognitive neuroscience.

**Table 1:** Conte Core Behavioral Test Battery.

**Beck Depression Inventory - II**^17^ A 12-item self-report questionnaire that examines depressive symptomatology over the prior 2 weeks. Total scores indicate level of depression: none, mild, moderate, severe.
**Empathizing Quotient**^13^ A 40-item self-report instrument that assesses the drive to identify others’ thoughts or emotions
**Mayer-Salovey-Caruso Emotional Intelligence Test (MSCEIT)**^16^ A computerized questionnaire that includes self-report items and emotion-identification tasks. Performance on eight subtests are combined to describe 4 aspects of emotion processing (perceiving, facilitating thought, understanding, and managing), which are further combined into two index scores (experiencing emotion and strategizing about emotion).
**Perceived Stress Scale**^77,78^ A ten-item self-response questionnaire that measures the extent to which a participant perceives personal life events in the previous month as stressful.
**Positive and Negative Affect Scales**^79^ A 20-item self-report measure designed to assess the current affective state.
**16 Personality Factors (16PF)**^80,81^ A self-report questionnaire comprised of 185 multiple-choice items addressing personal preferences and tendencies. The normative sample reflects the 2000 census data on age, sex, race, and education level. Scores reflect 5 global personality factors (Extraversion, Anxiety, Tough-Mindedness, Independence, and Self-Control), as well as 16 personality dimensions (primary scales) that are anchored by polarized characteristics. For example, scores on the “Warmth” factor reflect the subject’s interest in social contact by placing them on a continuum from “reserved” to “warm.” Other Primary Scales include: Reasoning (concrete vs. abstract), Emotional Stability (reactive vs. emotionally stable), Dominance (deferential vs. dominant), Liveliness (serious vs. lively), Rule-Consciousness (expedient vs. rule-conscious), Social Boldness (shy vs. socially bold), Sensitivity (utilitarian vs. sensitive), Vigilance (trusting vs. vigilant), Abstractedness (grounded vs. abstracted), Privateness (forthright vs. private), Apprehension (self-assured vs. apprehensive), Openness to Change (traditional vs. open to change), Self-Reliance (group-oriented vs. self-reliant), Perfectionism (tolerates disorder vs. perfectionistic), and Tension (relaxed vs. tense).
**Social Network Index**^82^ A self-report questionnaire used to quantify the extent of one’s social connections during a specific timeframe. Outcome variables include: a) Network Diversity (number of social roles in which the respondent has contact with one person or more at least once every 2 weeks; maximum is 12 including spouse, parent, child, child-in-law, close relative, close friend, church/temple member, student, employee, neighbor, volunteer, and group member), b) Number of People in Social Network (measures the total number of people which whom respondent maintains contact at least once every 2 weeks–reflecting overall network size), and c) Number of Embedded Networks (measures the number of different groups these contacts belong to, reflecting network complexity; maximum is 8, including family, friends, church/temple, school, work, neighbors, volunteering, and groups).
**Social Responsiveness Scale - Second Edition, Adult Form, Self-Report**^15^ A 65-item self-report questionnaire that assesses the presence of social difficulties common in autism.
**Systematizing Quotient - Revised**^14^ A 75-item self-report questionnaire that assesses the drive to understand and construct lawful systems for governing behavior.
**State Trait Anxiety Inventory (state & trait)**^54^ A self-report questionnaire that differentiates between the temporary condition of “state anxiety” and the more general and long-standing quality of “trait anxiety.” State anxiety is characterized by feelings of apprehension, tension, nervousness, and worry.
**Wechsler Abbreviated Scales of Intelligence (WASI)**^52^ A measure of cognitive abilities which includes 4 subtests (Matrix Reasoning, Block Design, Vocabulary, and Similarities) and provides 3 index scores (Full Scale IQ, Verbal Intelligence Quotient, and Performance Intelligence Quotient).
**Wechsler Abbreviated Scales of Intelligence - Second Edition (WASI-II)**^53^ A measure of cognitive abilities which includes 4 subtests (Matrix Reasoning, Block Design, Vocabulary, and Similarities) and provides 3 index scores (Full Scale IQ, Verbal Comprehension Index and Perceptual Reasoning Index).

Three features further distinguish our dataset: (1) we went to considerable lengths to control the quality of surface reconstructions by manual visual inspection and correction of all structural MRI datasets (where necessary); (2) we provide functional neuroimaging data in multiple preprocessed formats and anatomical spaces (including both volumetric and surface data) with open-source processing tools. This not only affords greater flexibility in how the data might be analyzed, but largely obviates the need to conduct further preprocessing or transformations by users — a task that can be complex and require substantial computational cost and time; (3) we provide a customizable denoising pipeline for the analysis of functional connectivity data that includes not only state-of-the-art denoising, but also incorporates the generation of functional connectivity matrices on the parcellated data and initializes analysis workflow for individual difference studies. Taken together, these features aim to provide a dataset that can most easily be used immediately to address scientific questions of interest by neuroscientists, psychologists, and data scientists.

## Methods

### Participants

Adults (enrollment n = 191; 18–50 years old at time of enrollment) were recruited from the Los Angeles area via Craigslist and publicly distributed flyers over the course of the past 8 years. Informed consent was obtained from all subjects prior to participation in accordance with the institutional review board (IRB) at the California Institute of Technology. Subjects were excluded if they had a full-scale IQ below 90, were not fluent in English, had a first-degree relative with schizophrenia or autism spectrum disorder, were currently taking psychotropic medication, had uncorrected vision or hearing impairment, and moderate-severe depression or indication of current suicidality (Beck Depression Inventory–II total = 25+; score of 3 or 4 on item 9^17^). Additional exclusionary criteria included history of any of the following: premature birth, epilepsy, major medical condition, metabolic disorder, chemotherapy or radiation, brain surgery, head injury, eating disorder, neurological condition, psychosis, bipolar disorder, autism, suicide attempt, substance dependence or abuse, alcoholism, color blindness or strabismus.

**Fig. 1:**
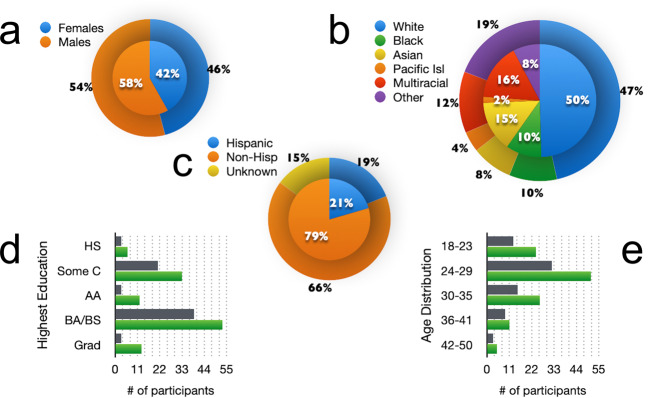
Demographics of Final Sample. Demographics of the final sample (n = 117, inner pie) are compared to demographics of participants who were excluded (outer pie; excluded n = 66; attrition n = 8). Top row: Sex (**a**) and Race (**b**) proportions. Middle: Ethnicity proportions (**c**). Bottom row: Number of participants by highest education level (**d**) and age grouping (e; green = final sample, gray = excluded/attrition group). Abbreviations: AA, Associates in Arts; BA/BS, Bachelor of Arts/Science; Grad, Graduate degree; HS, high school; Some C, some college.

Following a brief phone screening, 191 individuals came to Caltech for the enrollment visit. The final sample was reduced to 117 individuals due to exclusions and attrition. Information acquired during the enrollment visit resulted in exclusion of 47 based on our inclusion/exclusion criteria, 19 were excluded during MRI safety screening or due to features of MRI testing (6 due to claustrophobia, incompatible tattoos or pregnancy, 12 due to excessive motion during MRI scanning, 1 incidental structural abnormality per expert radiological review) and 8 dropped out of the study following the enrollment visit. The final participant group of 117 adults did not differ from the initial sample in gender (χ^2^ = 0.305, *p* = 0.581), age (*t*(187) = −0.594, mean difference = −0.581, 95% CI [−2.511, 1.349]), ethnicity (χ^2^ = 0.072, *p = *0.789), or race (χ^2^ = 4.270, *p* = 0.640) (Fig. 1).

### Structural magnetic resonance imaging

**Fig. 2:**
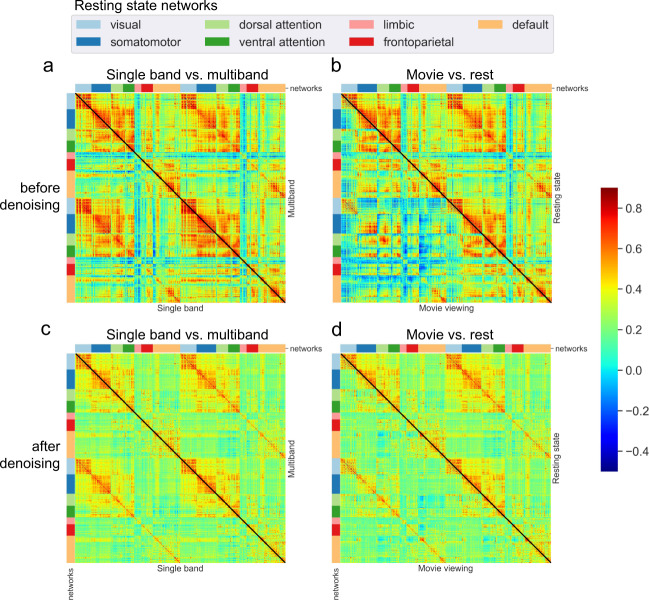
Comparison of functional connectivity (FC) matrices estimated before denoising (top row, **a,b**) and after denoising (bottom row, **c,d**) on subjects with two complete resting-state runs (N = 116). On the left (**a,c**), the lower triangular matrices are the average FC derived from single-band resting-state acquisitions (N = 34), while the upper triangular matrices show the average FC derived from multiband resting-state acquisitions (N = 100). Note that some subjects (N = 18) have both SB and MB scans and therefore contribute to both upper and lower triangles. On the right (**b,d**), lower triangular matrices are derived from movie fMRI data (N = 57), while upper triangular matrices are derived from multiband resting-state acquisitions (N = 100). We used data in CIFTI format registered to the MNI152NLin2009cAsym space, processed through fMRIPrep and denoised with rsDenoise with the strategy described in^48^ For each subject, two runs were concatenated before computing the average time series for each of 400 parcels of the Schaefer cortical parcellation^83^. Parcels are grouped following the 7 resting-state networks defined in the Yeo parcellation^84^. FC was computed as the pairwise Pearson’s correlation between parcel time series (color scale). For subjects with more than one session available, individual FC matrices are averaged across sessions before averaging them across subjects (so that each subject only contributed once).

All MRI data were acquired using either a 3 Tesla TIM Trio (2012 to 2017) or an upgraded 3 T Prisma.Fit system (2018 to 2019) (Siemens Medical Solutions, Malvern, PA) with a 32 channel head receive array coil. Stimulus presentation and response capture were performed using an LCD back-projection system and optical response button box (controlled via Psychophysics Toolbox 3). T1w structural imaging was performed in all 117 participants. T2w imaging was added in the second phase of the Conte Center (2018 onwards) and both structural contrasts were acquired in a subset of participants (Fig. 2). Incremental modifications were made over the years to the structural imaging protocol, including a change in spatial resolution from 1 mm to 0.9 mm isotropic, the addition of lipid suppression and a change in T1w pulse sequence from single-echo MP-RAGE to multi-echo MEMP-RAGE, which are summarized in Table 2.

**Table 2:** Structural and functional MRI sequence parameters for all protocol versions of the Caltech Conte Imaging Core.

T1w Structural									
Protocol Version	Sequence	Acquisitions	Total Time	Voxel (mm)	TR/TE (ms)	TI (ms)	Flip Angle (deg)	Fat Suppression	R
1.1	MP-RAGE	2	0:12:52	1.0 × 1.0 × 1.0	1500/2.9	800	10	None	1
1.2	MP-RAGE	2	0:12:52	1.0 × 1.0 × 1.0	1500/2.9	800	10	None	1
1.3.0	MP-RAGE	2	0:12:52	1.0 × 1.0 × 1.0	1500/2.9	800	10	None	1
1.3.1	MP-RAGE	2	0:12:52	1.0 × 1.0 × 1.0	1500/2.9	800	10	Water Excite	1
1.4	MP-RAGE	2	0:12:36	0.9 × 0.9 × 0.9	2400/2.6	1000	8	Water Excite	2
2.1	MEMP-RAGE	1	0:06:03	0.9 × 0.9 × 0.9	2530/Var	1100	7	None	2
2.1.1	MEMP-RAGE	1	0:06:03	0.9 × 0.9 × 0.9	2530/Var	1100	7	None	2
2.2	MEMP-RAGE	1	0:06:03	0.9 × 0.9 × 0.9	2550/Var	1100	7	Water Excite	2
**T2w Structural**									
***Protocol Version***	**Sequence**	**Acquisitions**	**Total Time**	**Voxel (mm)**	**TR/TE (ms)**	**TI(ms)**	**Flip Angle (deg)**	**Fat Suppression**	**R**
*1.1*	—	—	—	—	—	—	—	—	—
*1.2*	—	—	—	—	—	—	—	—	—
*1.3.0*	T2 SPACE	1	0:08:02	1.0 × 1.0 × 1.0	2500/144	—	Var	None	2
*1.3.1*	T2 SPACE	1	0:08:02	1.0 × 1.0 × 1.0	2500/144	—	Var	None	2
*1.4*	T2 SPACE	1	0:08:42	0.9 × 0.9 × 0.9	2500/212	—	Var	None	2
*2.1*	T2 SPACE	1	0:04:43	1.0 × 1.0 × 1.0	3200/390	—	Var	None	2
*2.1.1*	T2 SPACE	1	0:04:43	0.9 × 0.9 × 0.9	3200/393	—	Var	None	2
*2.2*	T2 SPACE	1	0:05:38	0.9 × 0.9 × 0.9	3200/564	—	Var	None	2
T2*w Functional MRI									
***Protocol Version***	***Sequence***	***Voxel (mm)***	***TR/TE (ms)***	***Flip Angle (deg)***	***Fat Suppression***	***EPI Echo Spacing (ms)***		***R***	***M***
1.1	GRE-EPI	3.0 × 3.0 × 3.0	2500/30	85	Yes	0.47		2	1
1.2	MB GRE-EPI	2.5 × 2.5 × 2.5	1000/30	60	Yes	0.54		1	4
1.3.0	MB GRE-EPI	2.5 × 2.5 × 2.5	1000/30	60	Yes	0.54		1	4
1.3.1	MB GRE-EPI	2.5 × 2.5 × 2.5	1000/30	60	Yes	0.54		1	4
1.4	MB GRE-EPI	2.5 × 2.5 × 2.5	1000/30	60	Yes	0.54		1	4
2.1	MB GRE-EPI	2.5 × 2.5 × 2.5	1000/30	60	Yes	0.54		1	4
2.1.1	MB GRE-EPI	2.5 × 2.5 × 2.5	1000/30	60	Yes	0.54		1	4
2.2	MB GRE-EPI	2.5 × 2.5 × 2.5	700/30	53	Yes	0.49		1	6
**B0 Fieldmap MRI**									
***Protocol Version***	***Sequence***	***Voxel (mm)***	***TR/TE (ms)***	***Flip Angle (deg)***	***Fat Suppression***	***EPI Echo Spacing (ms)***		***R***	***M***
1.1	Dual Echo GRE	3.0 × 3.0 × 3.0	400/2.6, 5.0	45	No	—		1	—
1.2	Dual Echo GRE	3.0 × 3.0 × 3.0	400/2.6, 5.0	45	No	—		1	—
1.3.0	Dual Echo GRE	3.0 × 3.0 × 3.0	400/2.6, 5.0	45	No	—		1	—
1.3.1	Dual Echo GRE	3.0 × 3.0 × 3.0	400/2.6, 5.0	45	No	—		1	—
1.4	MB SE-EPI	2.5 × 2.5 × 2.5	4800/50	90	Yes	0.54		1	1
2.1	MB SE-EPI	2.5 × 2.5 × 2.5	4800/50	90	Yes	0.54		1	1
2.1.1	MB SE-EPI	2.5 × 2.5 × 2.5	4800/50	90	Yes	0.54		1	1
2.2	MB SE-EPI	2.5 × 2.5 × 2.5	5500/48	90	Yes	0.49		1	1

MB: multiband, R: in-plane acceleration factor, M: multiband (MB) slice acceleration factor.

### Functional magnetic resonance imaging

High-quality BOLD fMRI data with whole-brain coverage were acquired in all subjects. BOLD resting-state and movie-viewing fMRI were acquired using single-band or multi-band 2.5 mm isotropic T2*-weighted EPI, depending on protocol version (Table 2 and Fig. 2). The imaging protocol was refined several times during the first phase of the Conte Center, but remained constant during the second phase following the scanner upgrade from TIM Trio to Prisma.Fit. Multiband acceleration was introduced in protocol version 1.2 and imaging parameters for all versions of the fMRI protocols are summarized in Table 2. Either dual-echo gradient echo imaging or phase-encoding polarity reversed SE-EPI image pairs were acquired for distortion correction immediately before each functional run, with identical slice geometry and EPI echo spacing to the BOLD EPI series.

Resting-state data consisted of two runs of between 400 (session 1p1) and 420 (session 2p2) seconds of resting-state with eyes open and instructions to fixate a white central cross on a black background. Movie viewing fMRI consisted of watching the black-and-white Hitchcock film “Bang! You’re Dead (1954)” and the short animated movie “Partly Cloudy”^18^. Alfred Hitchcock’s “Bang! You’re Dead (1954)” movie was edited from the original 20 min running time down to 8 min., as in^19^. Instructions were shown on the screen until the subject pressed a key, followed by 10 s of blank screen with a fixation cross. The movie played for 8-min, followed by 10 s blank screen with fixation cross until the end of scanning. The “Partly Cloudy” movie began after 10 s of rest (black screen; TRs 0–5). The first 10 s of the movie consisted of the opening credits (Disney castle, Pixar logo; 12–20 s), followed by 5 minutes, 14 seconds of the movie (without credits at the end), followed by 10 seconds of rest.

### Preprocessing of MRI Data

#### FreeSurfer segmentation and cortical parcellation

We performed cortical reconstruction and volumetric segmentation of T1w images outside of fMRIPrep with the FreeSurfer image analysis suite (version 7.1.0, http://surfer.nmr.mgh.harvard.edu/)^11,20–22^. In summary, processing included motion correction and averaging^23^ of volumetric T1w images, removal of non-brain tissue, automated Talairach transformation, segmentation of the subcortical white matter and deep gray matter volumetric structures, intensity normalization^24^, tessellation of the gray matter-white matter boundary, automated topology correction^25,26^, and surface deformation following intensity gradients for optimal tissue boundary placement. T1w MP-RAGE data were used for FreeSurfer reconstruction if T1w MEMP-RAGE data from the Phase 2 protocol were unavailable for a given subject. T2w images were passed to FreeSurfer reconstruction where available (n = 59). See Fig. 2 for a full breakdown of T1w and T2w image availability for all subjects.

#### Standardized MRI preprocessing

Both structural and functional MRI data were minimally preprocessed using fMRIPrep 20.2.1^10^, which is based on Nipype 1.5.1^27^. The processing steps for anatomical and functional MR data are summarized below, with specific software noted in italics. Independent, quality controlled FreeSurfer reconstructions (above) were integrated automatically by the fMRIPrep pipeline. Preprocessing scripts, including the exact parameters used with fMRIPrep and a detailed description of individual steps are provided in the code folder of the OpenNeuro BIDS data release^28^.

### Anatomical data preprocessing

T1-weighted (T1w) structural images were corrected for intensity non-uniformity (*N4BiasFieldCorrection, ANTS 2.3.3*)^29,30^ and skull-stripped (*antsBrainExtraction.sh, ANTS 2.3.3*). Brain tissue was segmented into cerebrospinal fluid (CSF), white-matter (WM) and gray-matter (GM) (*fast*, *FSL 5.0.9*)^31^. Where multiple T1w images were available for a given subject, a robust, registered average was constructed (*mri_robust_template, FreeSurfer 6.0.1*)^20^. Brain extracted T1w images were then registered diffeomorphically (*antsRegistration, ANTs 2.3.3*) to two standard spaces: (1) the ICBM/MNI 152 2009c Nonlinear Asymmetric space used by OpenNeuro (MNI152NLin2009cAsym)^32^ and (2) the ICBM/MNI 152 Version 6 Nonlinear Asymmetric space used by FSL (MNI152NLin6Asym)^33^.

#### Functional data preprocessing

For each of the BOLD runs found per subject (across all tasks and sessions), the following preprocessing was performed. First, a reference volume and its skull-stripped version were generated by aligning and averaging single-band references (SBRefs). Spatial distortion corrections for BOLD EPI data were derived from two spin echo EPI reference images with opposing phase-encoding directions (*3dQwarp, AFNI 20160207)*^34^. A distortion-corrected BOLD EPI reference image was constructed and registered to the T1w reference using a boundary-based approach (*bbregister, Freesurfer*)^35^. Rigid-body head-motion parameters with respect to the BOLD EPI reference were estimated (*mcflirt, FSL 5.0.9*)^36^ before any spatiotemporal filtering. BOLD runs belonging to the single band acquisition sessions were slice-time corrected (*3dTshift, AFNI 20160207*). The BOLD time series were resampled onto the fsaverage and fsaverage6 standard FreeSurfer surface spaces. The BOLD time series (including slice-timing correction when applied) were resampled onto their original, native space by applying a single, composite transform to correct for head motion and susceptibility distortions. The BOLD time series were resampled into the *MNI152NLin2009cAsym* standard space. Grayordinate files^37^ containing 91,000 samples were also generated using the highest-resolution fsaverage as an intermediate standardized surface space. Several physiological confound time series were calculated based on the preprocessed BOLD: framewise displacement (FD), DVARS and three region-wise global signals. FD was computed for each functional run using two definitions: absolute sum of relative motions^38^ and relative root-mean-square displacement between affine transforms^36^.

### Physiological Denoising of fMRI Data

Physiological noise regressors were extracted using CompCor and are provided for use in alternative physiological denoising approaches, but were not used in the rsDenoise pipeline described below^39^. Principal components were estimated for the two CompCor variants: temporal (tCompCor) and anatomical (aCompCor). A mask to exclude signal originating in cortex was obtained by eroding the brain mask, ensuring it only contained subcortical structures. Six tCompCor components were then calculated including only the top 5% variable voxels within that subcortical mask. For aCompCor, six components were calculated within the intersection of the subcortical mask and the union of CSF and WM masks calculated in T1w space, after their projection to the native space of each functional run. Framewise displacement^38^ was calculated for each functional run using the approach implemented by *Nipype*.

**Fig. 3:**
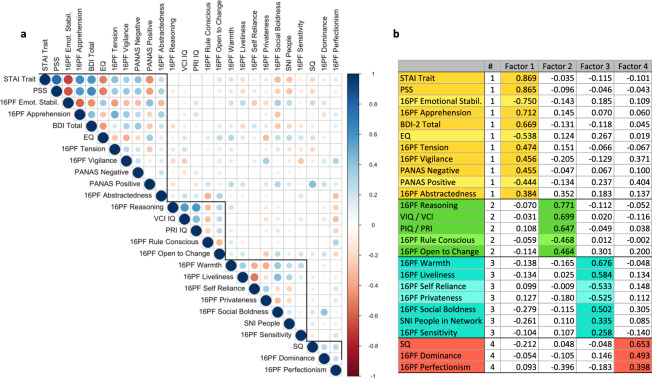
Factor Analysis. (**a**) Spearman rank-order correlations between each pair of variables. Variables are ordered according to the four-factor varimax-rotated solution, with dark outline boxing each factor grouping. (**b**) Four-factor varimax-rotated solution based on data from 144 participants. Maximal absolute loadings of task scores onto each of the four factors, leading to the interpretation of the factors we give in the text. Lighter colors indicate flipped scale interpretation (negative loadings).

Resting-state and movie fMRI data were further processed with *rsDenoise* (https://github.com/adolphslab/rsDenoise), a denoising pipeline specifically designed to correct for artifactual influences of non-neuronal fluctuations in signals acquired in the absence of an explicit task. This software was originally developed to study individual differences in intelligence and personality detectable from resting-state fMRI functional connectivity data^40,41^. The pipeline is based on open-source libraries and frameworks for scientific computing, including *SciPy, Numpy, NiLearn, NiBabel, Nipype, Scikit-learn, Pandas* and *Matplotlib*^27,42–47^, and accepts both volumetric data (in NIfTI format) and surface data (GIfTI or CIFTI format) that were minimally preprocessed with either fMRIPrep or the HCP pipelines^37^. It implements a wide variety of denoising strategies described by previous literature^1,48–51^, and works by performing a sequence of operations grouped in seven categories: motion scrubbing, voxel-wise normalization, detrending, tissue regression, global signal regression, motion regression and temporal filtering. In addition to enabling the user to reproduce previously published methods, the software allows testing of new combinations of denoising steps and adding custom functions to the pipeline. The pipeline also offers support for the generation of functional connectivity matrices (as in Fig. 3) and a framework for the prediction of individual differences from functional connectivity features. For the results presented in this work, we adopted a pipeline that reproduces the denoising strategy described in^48^. There are seven consecutive steps: (1) each voxels’ signal is z-score normalized, (2) using tissue masks, temporal drifts from cerebrospinal fluid (CSF) and white matter (WM) are removed with third-degree Legendre polynomial regressors, (3) CSF and WM mean signals are regressed from gray matter (GM) voxels, (4) rotational and translational realignment parameters and their temporal derivatives are used as explanatory variables in motion regression, (5) signals are low-pass filtered with a Gaussian kernel, (6) temporal drift from gray matter (GM) signal is removed using third-degree Legendre polynomial regressors, and (7) lastly global signal regression (GSR) is performed.

### Behavioral assessment

Assessment of cognitive and behavioral functioning was conducted using the 12 standardized psychological instruments described in Table 1. These instruments were administered by one trained research assistant (T.A.), and the majority of data were collected on one day. Demographic and behavioral data are curated in a comma-separated value (CSV) file, accompanied by a data dictionary explaining all variables^28^. The dataset includes summary scores and item-wise responses. Descriptive group statistics of the summary scores from all behavioral measures are provided in Table 3.

**Table 3:** Summary of Behavioral Data.

Index/Scale	N	Mean	SD	Min	Max	Mean Diff	95% CI	
FSIQ (standard score, *μ* = 100)	117	106.89	9.65	87	132	**6.889**	5.165	8.571
PIQ/PRI	117	103.78	10.67	83	133	**3.778**	1.821	5.762
VIQ/VCI	117	108.03	9.75	87	137	**8.034**	6.323	9.769
BDI-2	117	5.03	5.30	0	25			
Empathizing Quotient	117	49.50	12.79	20	74			
MSCEIT (standard score, *μ* = 100)								
Perceiving	97	105.21	15.26	70	150	**5.213**	2.294	8.232
Using	97	100.74	13.47	64	127	0.736	−1.802	3.437
Understanding	97	101.40	9.86	72	126	1.400	−0.649	3.312
Managing	97	96.59	9.28	70	114	**−3.410**	−5.255	−1.458
Perceived Stress Scale	117	12.46	6.65	0	32			
PANAS								
Positive	117	32.40	8.65	12	50			
Negative	117	12.46	4.23	10	33			
16PF (sten, *μ* = 5.5)								
Warmth	117	5.02	1.65	1	9	**−0.483**	−0.786	−0.175
Reasoning	117	5.48	1.79	1	9	−0.021	−0.338	0.307
Emotional Stability	117	5.23	1.56	1	8	−0.269	−0.548	0.030
Dominance	117	5.27	1.68	2	9	−0.226	−0.554	0.084
Liveliness	117	6.38	1.79	3	9	**0.876**	0.518	1.214
Rule-Consc.	117	4.00	1.49	1	7	**−1.500**	−1.779	−1.241
Social Boldness	117	5.96	1.82	2	9	0.457	0.156	0.773
Sensitivity	117	6.21	1.51	3	10	**0.705**	0.407	0.973
Vigilance	117	6.46	1.81	3	10	**0.962**	0.647	1.318
Abstractedness	117	6.33	1.68	2	10	**0.833**	0.527	1.152
Privateness	117	5.53	1.94	1	9	0.030	−0.324	0.372
Apprehension	117	5.39	1.77	1	9	−0.107	−0.421	0.203
Open to Change	117	6.74	1.55	2	10	**1.235**	0.959	1.500
Self-Reliance	117	6.15	1.77	2	10	**0.645**	0.337	1.000
Perfectionism	117	5.28	1.57	1	9	−0.218	−0.526	0.056
Tension	117	5.03	1.73	2	9	**−0.466**	−0.787	−0.150
Social Network Index								
Network Diversity	117	4.54	1.65	0	9			
Total People	117	15.21	12.68	0	106			
Embedded Networks	117	1.38	1.24	0	5			
SRS- 2 Adult, SR (T-score, *μ* = 50)	99	49.80	8.04	36	75	−0.202	−1.585	1.494
State Trait Anxiety Inventory (T-score, *μ* = 50)								
Trait	117	49.06	9.81	33	87	−0.940	−2.736	0.893
State	117	45.15	8.04	34	73	**−4.855**	−6.263	−3.386
Systematizing Quotient	117	67.88	20.81	22	138			

Bold indicates the 95% confidence interval of the difference between expected mean and means of 1000 bootstrapped samples did not include zero. Mean Diff = mean difference from the expected mean (e.g. participant T-score minus 50); SD = standard deviation; 95% CI = 95% confidence interval of the mean difference based on 1000 bootstrapped samples; SRS-2 = Social Responsiveness Scale - 2; SR = Self-report; Consc. = Conscientiousness.

Descriptive group statistics of the summary scores from all behavioral measures are provided in Table 3. When available, participants’ scores were converted to standardized scores using published norms that account for demographic factors relevant to each measure (per the publisher). The WASI^52^, WASI-II^53^, and SRS-2^15^ norms are age-specific. STAI^54^ and MSCEIT^16^ norms are specific for age and sex. Table 3 presents 95% confidence intervals for the difference from the expected mean (e.g. participant T-score minus 50) based on 1000 bootstrapped samples. The 95% confidence intervals indicate that our cohort had elevated IQ scores, with elevated emotion perception but lower emotion management (MSCEIT) scores than the published normative sample. On average, personality traits (16PF) reported in our sample indicated elevations in liveliness, sensitivity, vigilance, abstractedness, openness to change, and self-reliance, with reduced evidence of warmth, rule-consciousness and tension. The 95% confidence intervals for SRS-2 and STAI trait anxiety difference scores included zero, but our cohort reported notably low levels of state anxiety. Additionally, for the tests with standardized scores we examined the number of participants who scored more than 1.5 standard deviations above or below the normative mean (i.e. within the range of clinical significance). After applying measure-wise Bonferroni adjustment, the frequency of participants with clinically-significant scores was not greater than expected by chance for any measure.

**Fig. 4:**
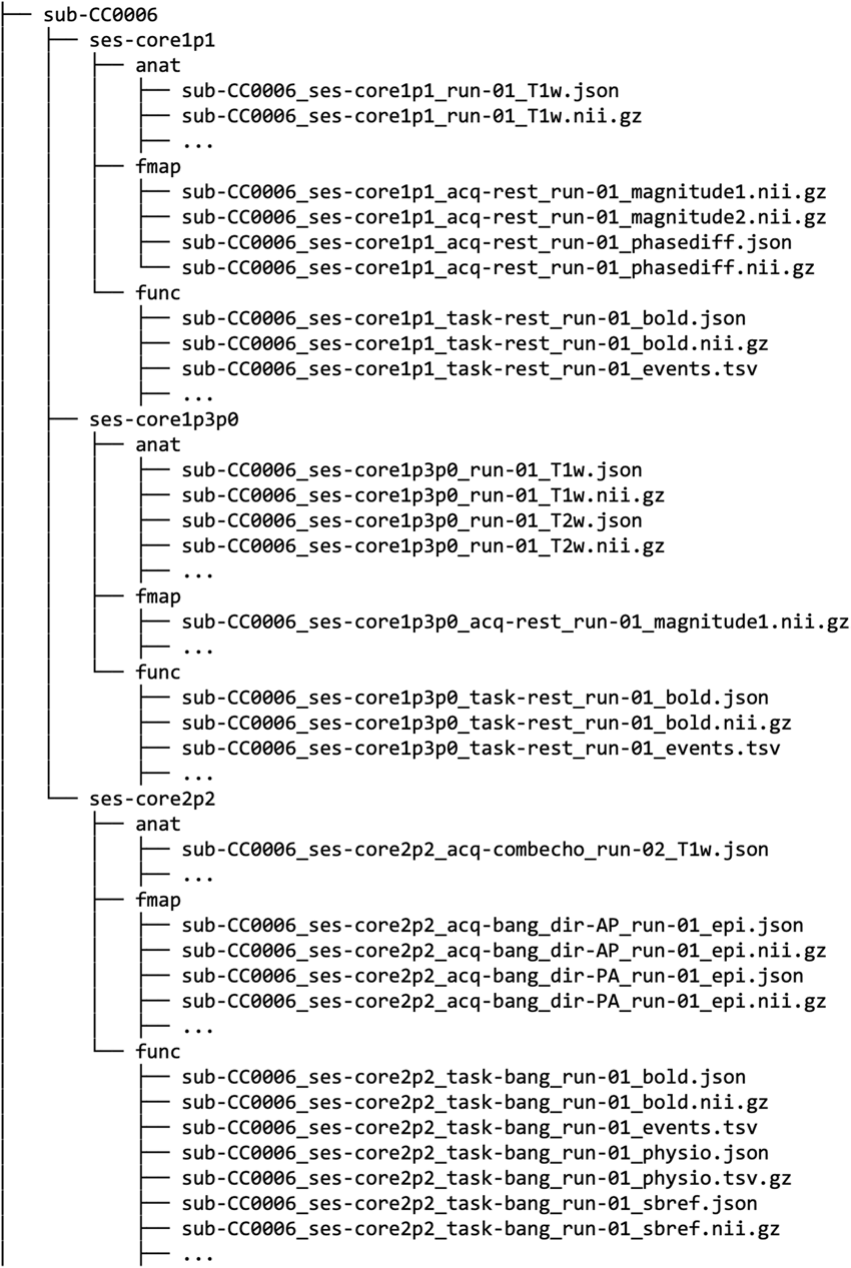
An example abbreviated BIDS directory structure for one subject showing the range of imaging and auxiliary data types available for multiple protocol variants. Briefly, the main data records consist of: (i) structural MRI (raw T1w and T2w images; manually edited segmented and parcellated cortical data), (ii) resting-state fMRI (raw, preprocessed, denoised, available in 3 anatomical spaces), (iii) movie fMRI (raw, preprocessed, available in 3 anatomical spaces), (iv) physiological data to accompany the fMRI datasets An overview of all the MRI data available across the entire subject sample is provided in Fig. 5.

In addition to the psychological variables from specific tasks, we also provide an example use case of the rich psychological data in an exploratory factor analysis based on all of the behavioral measures available in all of the subjects (Note: MSCEIT and SRS-2 were not included as they were not available for all participants and STAI state was not included due to high correlation with STAI trait; Fig. 4). We conducted exploratory factor analysis on all subjects with complete datasets, which were 144 Conte Center participants, of which the 117 whose imaging data are presented here were a proper subset. Due to non-normal distribution of multiple measures, Spearman rank-order coefficients were used for all correlations in the factor analysis (see Fig. 4). The number of factors was estimated in R^55^ using the following methods (processing packages are shown in italics): Horn’s Parallel Analysis^56^ (*paran*); Cattell’s Scree Optimal Coordinate Index^57^ (*nFactors*); CNG scree test^58^ (*nFactors*); Zoski and Jurs’ multiple regression b coefficient^59^ (*nFactors*); the Minimum Average Partial (MAP) test, both the original^60^ and revised^61^ versions (*paramap*); and the Very Simple Structure criterion (*vss*). All tests, with the exception of Horn’s Parallel Analysis, consistently predicted three to four factors. Based on these estimates, four factors were retained. The R code for estimating the optimal number of factors and generating rotated and unrotated solutions for 3- and 4-factor models, as well as all data files related to this analysis are provided at https://github.com/adolphslab/ConteDataRelease/blob/main/FactorAnalysis/Factor_Analysis.R.

Specifying a three-factor and four-factor solution, factor analysis was conducted in R using maximum likelihood estimation, with varimax rotation and without rotation (*fa*), and factor scores were generated with the Bartlett formula. Figure 4 shows factor loadings for the four-factor varimax-rotated solution. Factor loadings for the rotated and unrotated solutions were highly congruent (r_c_ = 0.99 for factors 1 and 2 and r_c_ = 0.91 for factors 3 and 4). Factor 1 is associated with negative emotionality, including elevations in anxiety, depression, stress, negative affect, and emotional instability, as well as lowered empathy. Factor 2 reflects cognitive flexibility, with elevations on cognitive ability and openness to change, and a negative association with rule consciousness. Factor 3 relates to elevated levels of social engagement. Factor 4 reflects cognitive rigidity. It is noteworthy that lowest factor loadings were for two social measures (SNI People in Network and 16PF Sensitivity), suggesting that while these factors account for some shared variance in social skills, they are unlikely to mask unique individual variations in social functioning. Individual scores across these 4 factors are provided for all our 117 subjects as part of this data release^28^; however, this is only one illustrative approach to factor analysis and should not preclude exploration using alternative methods.

## Data Records

**Fig. 5:**
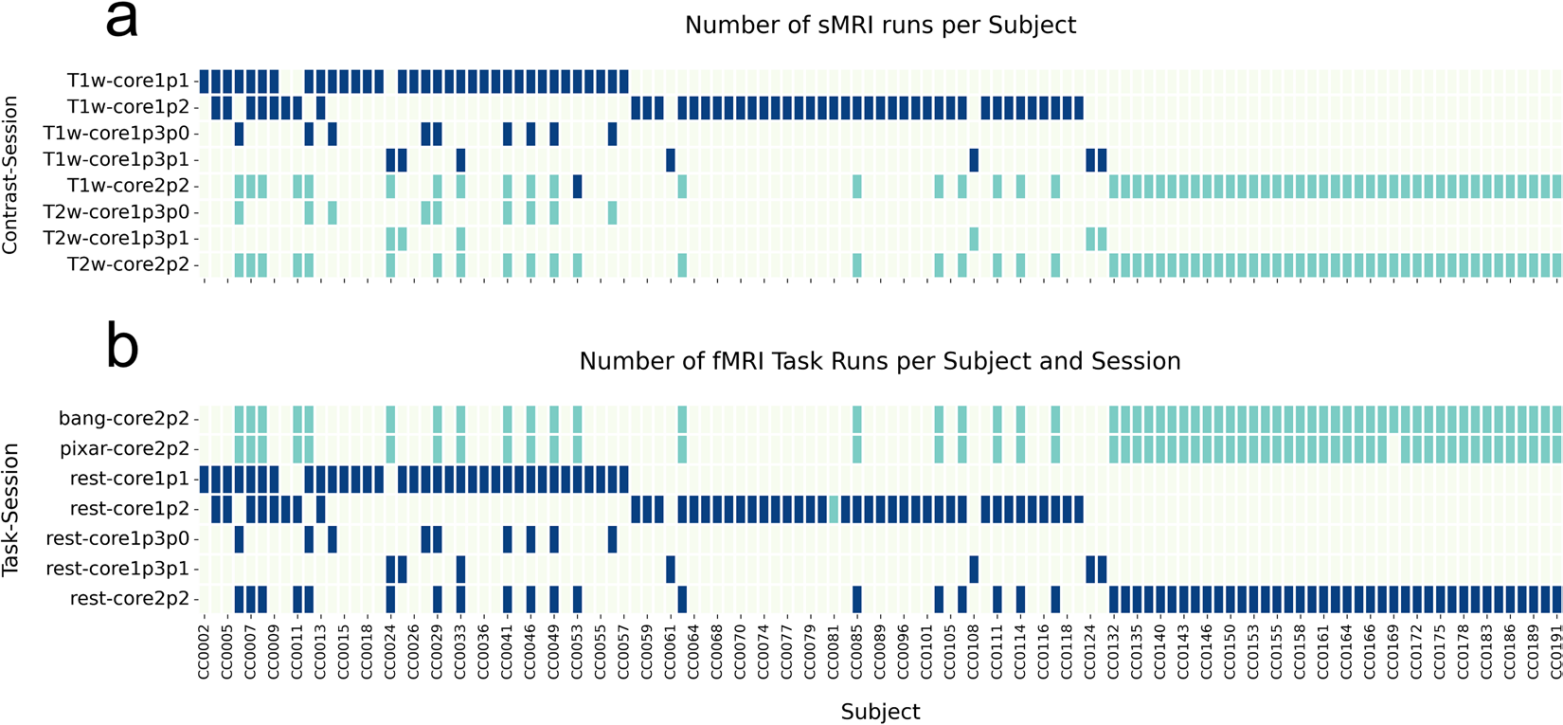
Availability of structural (sMRI, a) and functional (fMRI, b) runs for each subject and session (i.e., protocol version). Note that not all subject ID labels are shown for clarity. Key: Cyan = one run, Blue = two runs. See Table 2 for full pulse sequence parameter details.

The data types described below are available on the OpenNeuro data sharing platform^28^. The dataset follows the Brain Imaging Data Structure (BIDS version 1.6.0)^9^ which organizes the imaging data using a simple folder structure with nested files, each with standardized file naming conventions and accompanying JSON and TSV format metadata. T1w and T2w structural images were irreversibly deidentified using a customization of *pydeface* (https://github.com/jmtyszka/voxface). An example of the data structure and variety of data types available for subjects is given in Fig. 5. Note that events TSV files are empty placeholders for BIDS validation in the absence of response behavior for passive movie viewing and resting-state series.

## Technical Validation

### Quality Control of Automated Cortical and Subcortical Reconstructions

**Fig. 6:**
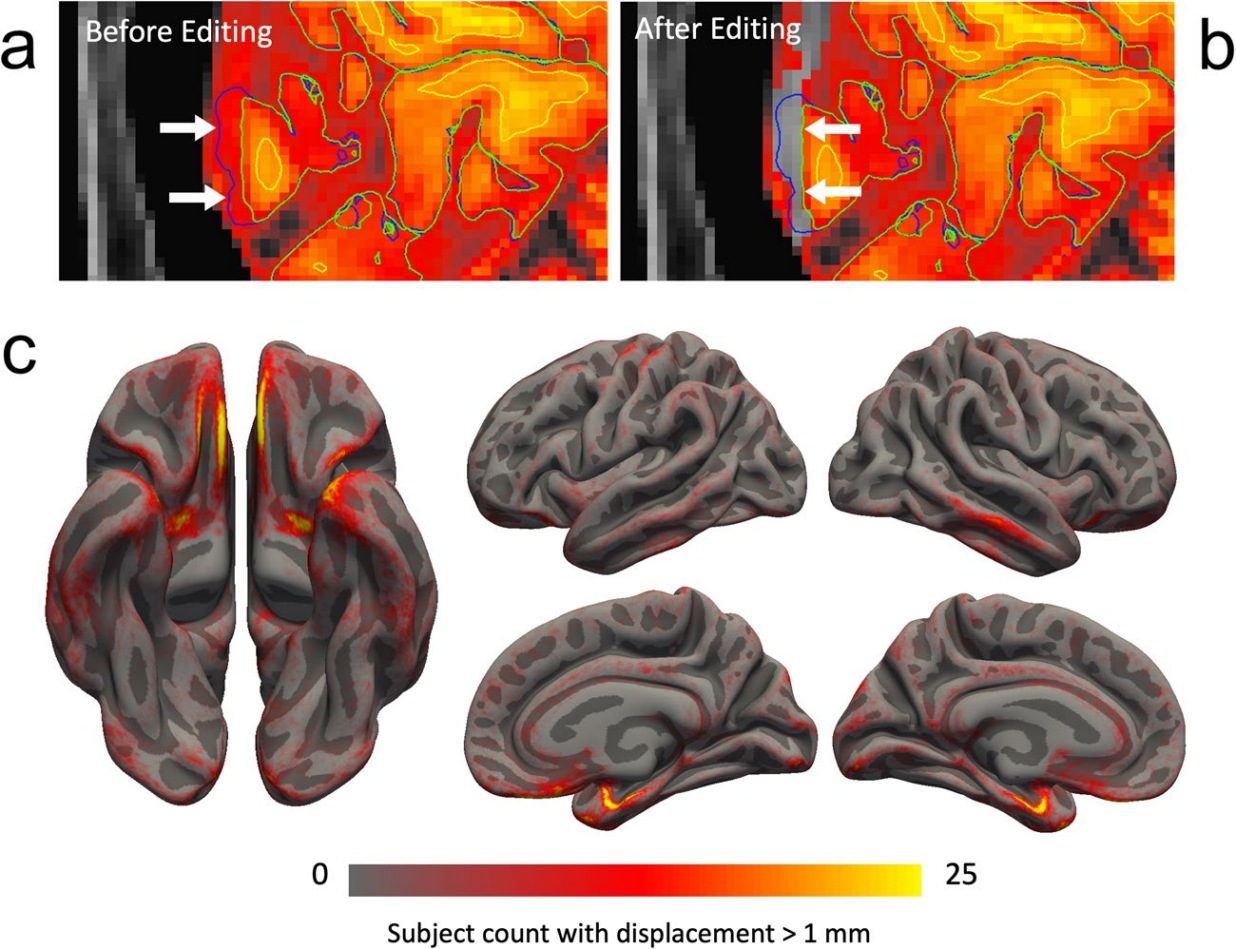
Example impact of manual brain mask editing on pial surface estimation. Prior to correction (**a**), the automatically estimated pial surface extended into the sagittal sinus (arrows). Deletion of voxels from the brain mask (**b**, heatmap color scale overlay) restored the pial surface to its edited position (arrows). (**c**) Cortical regions requiring pial surface editing. The number of subjects with pial surface displacement following editing of greater than 1 mm is shown overlaid on the partially inflated fsaverage pial surface. Overall, pial editing is concentrated in medial temporal, ventromedial frontal and lateral temporal cortices, consistent with areas prone to local susceptibility effects resulting in boundary inaccuracies.

Freesurfer supports visual inspection and manual corrections of automatic reconstruction to the initial and final brain masks, white and gray matter delineation and specification of white matter bias correction control points. All initial tissue constructions were visually inspected and manually corrected as necessary by a team of eight trained editors (DK, DAK, TR, ZE, DL, SL, WZ, JMT). Training included i) prior training through Freesurfer course material and ii) expert-guided learning of manual interventions (http://surfer.nmr.mgh.harvard.edu/fswiki/CourseDescription). Editors were randomly assigned to edit 10–15 scans. The most common issues that needed correction included: 1) inclusion of non-brain tissue (e.g., dura, skull, sinus blood) in the grey matter (pial surface), 2) incomplete temporal pole reconstruction, 3) white matter surface inaccuracies in ventral temporal regions. Manual edits were applied as outlined in detail by the FreeSurfer documentation (http://surfer.nmr.mgh.harvard.edu/fswiki/Tutorials) and respective reconstruction steps were run as implemented by the pipeline. Resulting next round reconstructions were again visually inspected and edited where necessary. An example of the impact of editing the brain mask on the pial surface in an individual subject is shown in Fig. 6 (top) with the surface displacement caused by editing, averaged over all subjects, shown in Fig. 6 (bottom).

### Quality control of fMRIPrep reports

fMRIPrep provides visual quality assessment reports per subject allowing a thorough visual assessment of processing quality. Three raters (D.K., J.M.T., P.G.) each visually inspected about one third of all reports, using previously agreed-upon criteria with regards to i) visual artifacts, ii) registration/transformation errors, iii) brain tissue segmentation and iv) quality of susceptibility distortion correction. We used a threshold intended to be conservative for gross errors, yet not specific to minor inaccuracies. We provide the three-tiered ratings (1, major issues; 2, minor issues; 3, no obvious issues) in a CSV file (fmriprep_output_manualQA.csv)^28^.

### Image quality control metrics for bold fmri

Detailed image quality metrics (IQMs) were calculated for all structural and functional imaging series using MRIQC (v0.15, Stanford Center for Reproducible Neuroscience)^62^ and full reports are included in this data release. Two example IQMs for the fMRI series, frame wise displacement (FD) and temporal signal-to-noise ratio (tSNR), are reported in more detail here.

### Framewise displacement

**Fig. 7:**
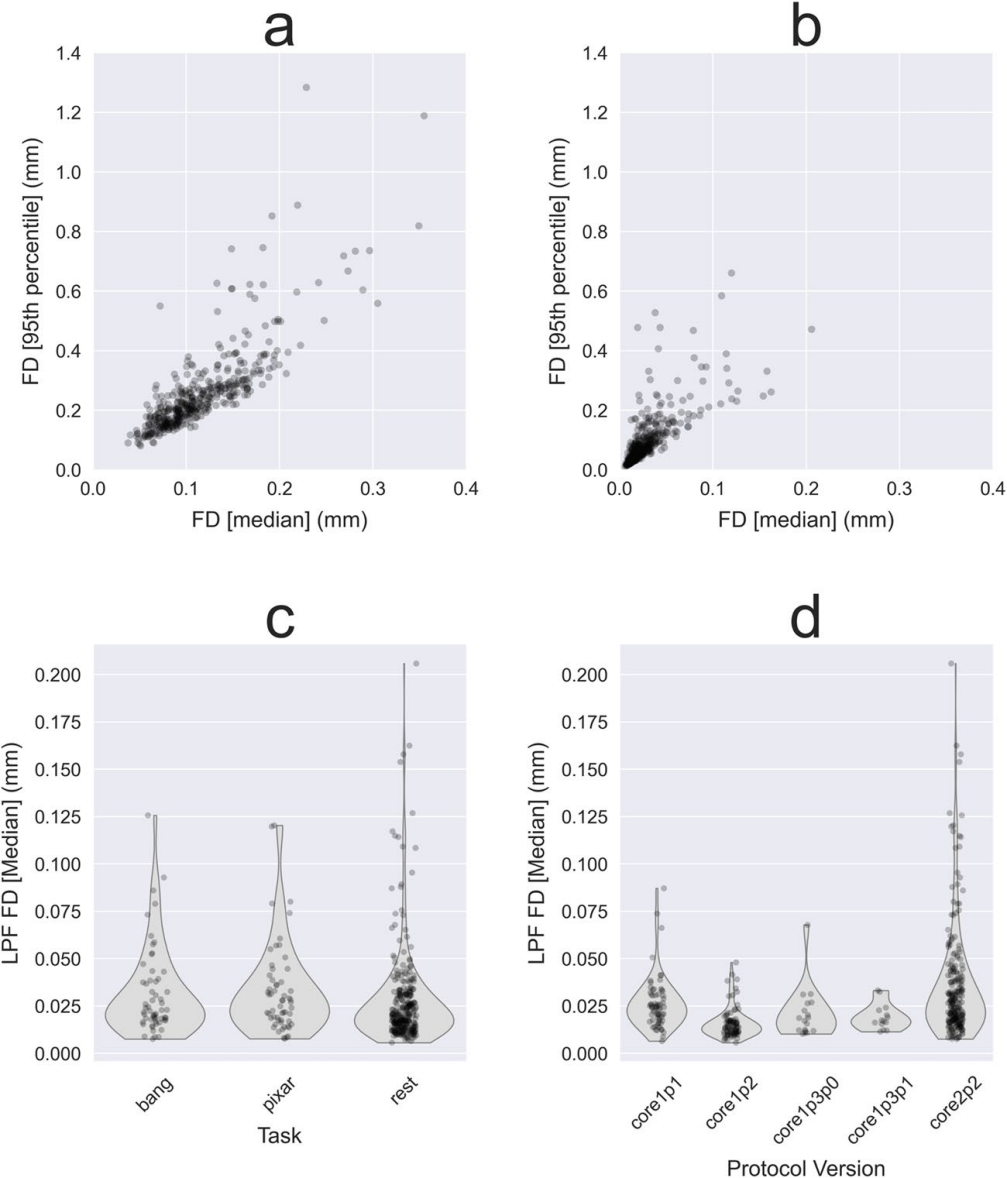
Head motion measured by framewise displacement (FD). (**a**) Raw and (**b**) low-pass filtered (LPF) temporal mean FD for all task and resting-state fMRI runs. The scatter plots compare typical (temporal median) and upper range (temporal 95th percentile) FD for all fMRI runs. (**c**) Temporal median LPF FD by task (“Bang, You’re Dead!”, “Partly Cloudy”) and resting-state, and (**d**) by MRI protocol version.

Rigid body head motion was characterized using the framewise displacement (FD) metric defined in^63^. FD was computed with and without linear low-pass filtering (LPF) (Butterworth filter, order 5, f < 0.2 Hz) of the individual motion parameter time series calculated by MRIQC. LPF minimizes high frequency respiratory contamination in FD timeseries following arguments made in^64–67^. Filtered FD distributions for the three fMRI experiments (“Bang, You’re Dead!”, “Partly Cloudy” and resting-state) are shown in Fig. 7. Note that a very small number of subjects show rare relatively large motion spikes at times during the scan, as expected in a larger sample. All motion is fully characterized in the combination of fMRIPrep and MRIQC reports of this data release^28^.

### Temporal SNR

**Fig. 8:**
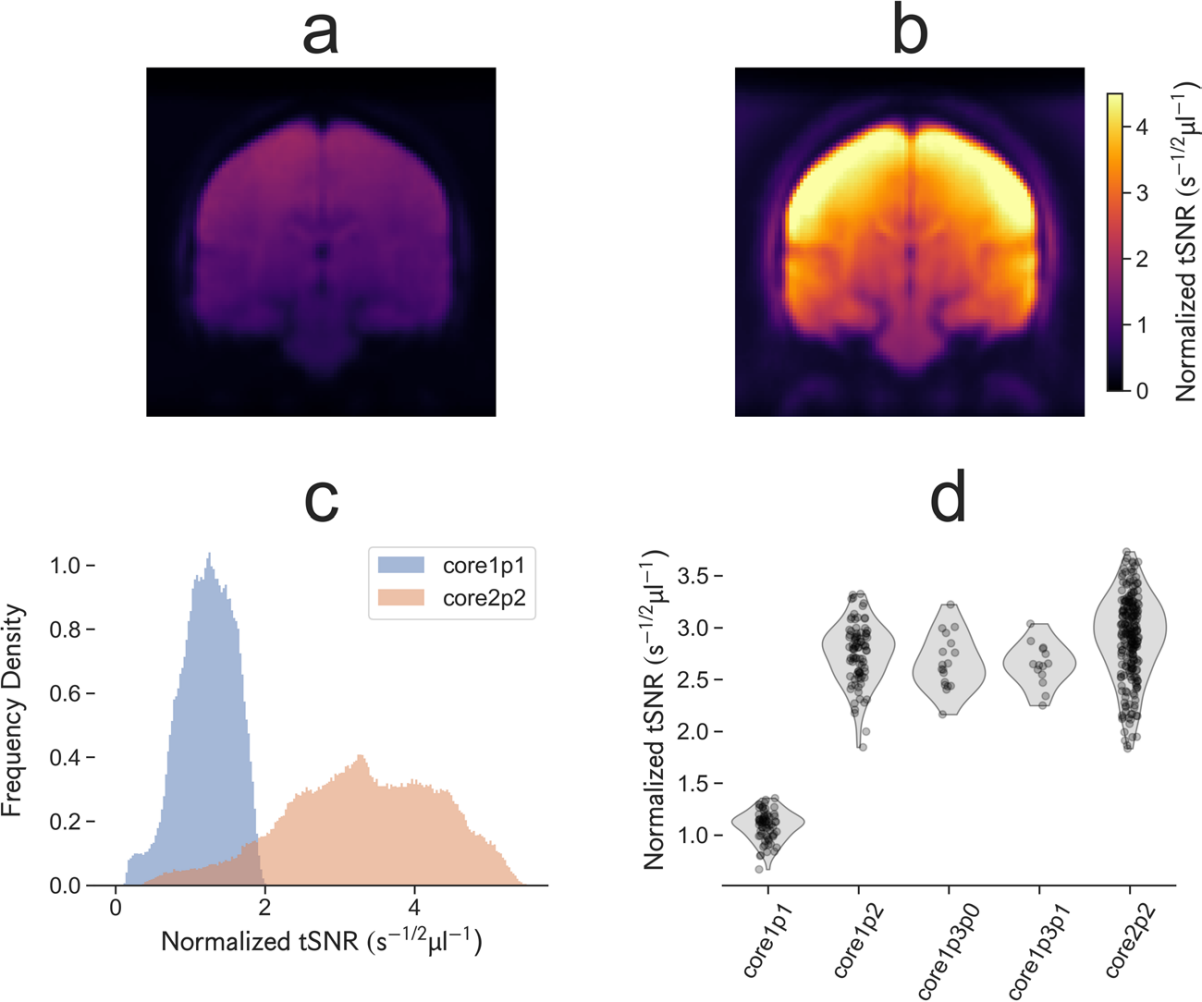
Mean, whole-brain temporal SNR normalized to repetition time and voxel volume for comparison between sequence protocol variants. Multiband protocol variants consistently perform between 2.5 and 3 times better than the single band variant (core1p1) in terms of tSNR efficiency. (**a**) Mid-coronal sections of the normalized tSNR efficiency (raw tSNR calculated by the MRIQC pipeline, adjusted for voxel volume and TR) averaged over all available subjects for the initial single-band protocol (core1p1) and (**b**) second phase multi-band T2*-weighted EPI protocol (core2p2) demonstrating the increase in normalized tSNR efficiency offered by multiband acquisitions despite the reduction in spatiotemporal resolution from 3.0 mm and 2.5 s to 2.5 mm and 0.7 s. (**c**) Normalized tSNR distributions within the brain, showing an approximately three-fold increase in mean normalized tSNR with the core2p2 protocol. (**d**) Whole-brain averaged normalized tSNR distributions for each task and protocol version.

Temporal signal-to-noise ratio (tSNR) was calculated by MRIQC for each fMRI series. Raw tSNR estimates were normalized to voxel volume and EPI repetition time (TR) to allow comparison between sequence variants with different multiband acceleration factors and spatial resolutions (Fig. 8).

## Usage Notes

### Limitation and opportunities of an in-depth sample of small size

As compared to other multimodal data releases such as the HCP or UK BioBank, the sample size of the present release is small. It is by now well known that small sample sizes severely limit the statistical reliability of conclusions that can be drawn about individual differences using neuroimaging data^68–70^, in line with a general upwards correction for the statistical reliability of correlations between datasets^71^. Generalization of findings regarding individual differences is thus limited in our dataset, although the details will vary depending on the exact question asked and method used^72^. As we have recommended previously^70^, we encourage the use of a predictive framework (using cross-validation within the dataset and/or replication to other, independent datasets), permutation-based statistical evaluation, and where feasible pre-registration in order to minimize the risk of false positive findings. A recent example based on a subset of the present dataset (prior to its further processing and release) illustrates that valuable negative findings, as well as estimates of sample sizes required for future studies, can be derived from this dataset^73^. We would anticipate that the present data release may be more valuable for adding cautionary notes and power estimates to the literature than for strong demonstrations of positive findings.

Nonetheless, the dataset is distinguished by its in-depth and comprehensive psychological and behavioral assessments, especially in the domain of social cognition and decision making. We note that the factor analysis that we also provide (Fig. 4), while of interest in its own right, in no way precludes more fine-grained investigation of the original individual variables. Indeed, we would recommend that the factors be considered as broader covariates in analyses that wish to isolate variance in a specific individual behavioral variable more selectively. The in-depth behavior data together with high-quality neuroimaging data provides a powerful platform to discover new brain-behavior relationships even with our modest sample size, since the measurement error of the variables is no less important than the sample size. However, we would expect such positive discoveries to be relatively constrained, ideally driven by specific pre-registered hypotheses. One possible research program could thus consist of an initial discovery study in a large-sample database, such as the UK Biobank, followed with a hypothesis-driven replication of the finding in our database—where the relevant variables are provided both with greater precision and, for the behavioral data, likely greater validity. The breadth of psychological characterization in our data release (see Table 1) provides further opportunities for comparison with other databases, where related cognitive variables are estimated from less detailed assessments. Applications of “far replicability”^74^ could be extended to databases in clinical populations (e.g., of participants with psychiatric diagnoses of depression, anxiety, autism, schizophrenia, and other disorders that impact social cognition and decision-making).

Our data release is also distinguished by providing multiple data formats and degrees of preprocessing. This affords the opportunity to test results, for instance, against variations in denoising decisions in an accessible and straightforward manner, as a further check on the robustness of findings to variations in typically complex processing pipelines, a well-known source of variation in the results obtained^75,76^. The denoising code we are co-releasing, in particular, allows researchers to explore a range of processing pipelines with substantial flexibility. Taken together, the internal processing flexibility enabled by this data release, together with the above recommendations to interface the present data with other datasets that purport to measure similar variables, should aim to maximize the meaningful generalizability of findings.

#### Note on the informed usage notes and quality control (QC)

We highlight below some processing and quality-specific aspects regarding the MRI data of this release.

We have used a combination of manual (human) and automated quality inspection of both structural (human: manual visual inspection and editing of FreeSurfer outputs; automated: MRIQC) and functional MRI data (human: manual visual inspection and resulting QC rating data; automated: MRIQC; see fMRIPrepQC_ratings.csv). We provide the outputs of our careful QC with the actual data resulting from it. It is the responsibility of the end-users to use the information available depending on their intended use of the data and study-specific QC criteria. For example, rigor and attention to minor surface reconstruction errors might be less strict for studies that aim to use cortical reconstruction outputs only for surface-based registration. In contrast, for a specific volumetric study (e.g., cortical thickness analysis), one might be less lenient. Note that given *in vivo* data (as well as the current possible imaging resolution) there is no clear “ground truth” for anatomical tissue segmentations, beyond consensus in human judgment of the images. In addition, remaining image quality aspects due to factors such as motion and regional susceptibility effects (e.g., in inferior temporal brain regions) cannot be eliminated *post hoc* and result in residual imprecision in individual data. These and other intrinsic measurement errors in our dataset require users to apply expert judgment in how they use the data release to answer specific scientific questions of interest.

For example, caution should be applied when using functional data in orbital frontal regions and data processed with fMRIPrep. As of the submission date of this paper, there is a known issue with susceptibility distortion correction (SDC) using spin echo fieldmaps as implemented in fMRIPrep. fMRIPrep currently uses AFNI’s *3dQwarp* function to implement distortion correction, which can produce suboptimal SDC outputs in some subjects (see https://github.com/nipreps/fmriprep/issues/2210). While issues such as this are not a result of our specific data, they can be serious issues that require knowledge about the limitations inherent to MRI and established processing tools, an ongoing set of issues actively discussed among expert users.

## Supplementary information

Supplementary Information

## Data Availability

We used containerized versions of fMRIPrep 20.2.1 and MRIQC for data preprocessing and quality control. Example calling scripts for fMRIPrep, jupyter lab notebooks for figure recreation and R code for the example factor analysis are provided at https://github.com/adolphslab/ConteDataRelease. The code to reproduce resting-state and movie analyses are provided at https://github.com/adolphslab/rsDenoise. As outlined in detail in the source, this codebase can easily be adapted to run many different configurations of denoising decisions on the data.
