# Supplementary material for: Caltech Conte Center, a multimodal data resource for exploring social cognition and decision-making
Source: Sci Data. 2022 Mar 31;9:138. doi: 10.1038/s41597-022-01171-2 (PMC8971509; doi:10.1038/s41597-022-01171-2)

**Supplementary Information**

**Figure SI1:** Author Contributions. Contribution strengths are indicated numerically: 0 (no contribution), 1 (support), 2 (medium), 3 (lead); and color-coded with darker colors indicating stronger contributions.


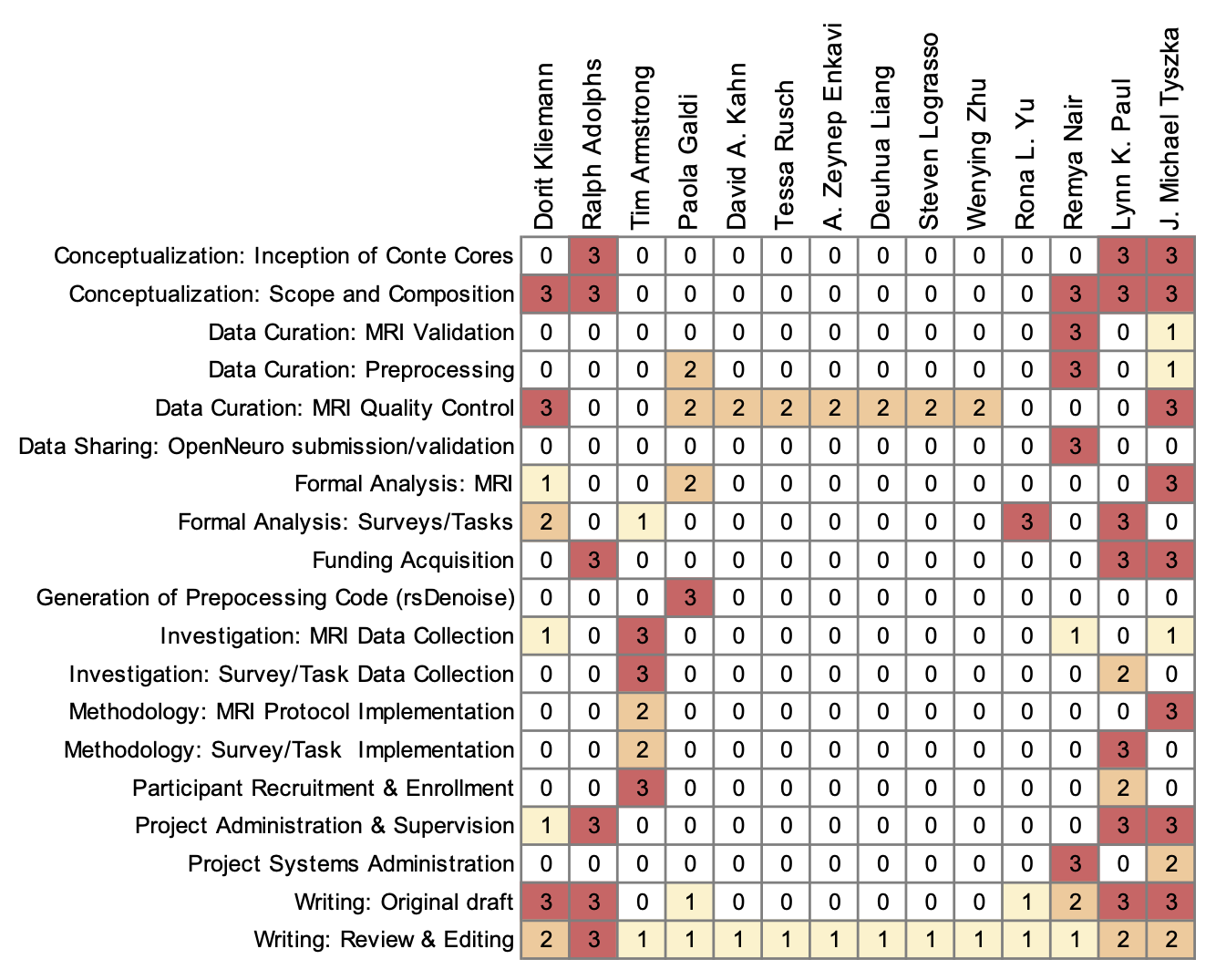

Supplement: Supplementary file 1 — Supplementary Information [file 41597_2022_1171_MOESM1_ESM.docx]
